# Primary biliary non-Hodgkin's lymphoma: A case report

**DOI:** 10.1097/MD.0000000000026110

**Published:** 2021-06-04

**Authors:** Jiamei Wu, Yin Zhou, Qingshu Li, Jing Zhang, Yun Mao

**Affiliations:** a Department of Radiology; b Department of Pathology, the First Affiliated Hospital of Chongqing Medical University, Chongqing, China.

**Keywords:** biliary tract, case report, cholangiocarcinoma, cholangitis, non-Hodgkin's lymphoma

## Abstract

**Rationale:**

Primary biliary non-Hodgkin's lymphoma (PBNHL) is a rare disease with only 41 cases reported since 1982. The incidence of PBNHL in patients with malignant cholangiocarcinoma was 0.6%, and PBNHL accounted for 0.4% of extranodal non-Hodgkin's lymphoma, and only 0.016% of all non-Hodgkin's lymphoma cases.

**Patient concerns:**

We present a rare case of PBNHL in a 59-year-old female who had jaundice for 3 days with weight loss and Epstein-Barr virus infection. Initial computed tomography and magnetic resonance imaging showed diffuse thickening wall of bile ducts with corresponding lumen stenosis, blurred fat space around the portal vein, lymphadenopathy, and a normal spleen. These manifestations and images were similar to hilar cholangiocarcinoma. So, the diagnosis of hilar cholangiocarcinoma was initially considered.

**Diagnoses:**

Postoperative pathology confirmed the final diagnosis was PBNHL.

**Interventions:**

The patient and her family requested to clarify the histologic diagnosis by laparotomy biopsy. Because the biopsy result could not be defined during operation, then right hemihepatectomy and choledochojejunostomy were performed. She did not receive any antitumor treatment.

**Outcomes:**

One month after the patient's first examination, both computed tomography and magnetic resonance images showed diminished stenosis of common bile duct and left hepatic duct, but a new mass in segment IV of liver was observed. Unfortunately, the patient died due to disease progression.

**Lessons:**

This case reminds us that although PBNHL is rare, making accurate diagnosis difficult preoperatively, PBNHL should be considered when encountering a case with Epstein-Barr virus infection and those typical imaging findings.

## 1 Introduction

Primary biliary non-Hodgkin's lymphoma (PBNHL) is a rare disease with only 41 cases reported since 1982.^[1]^ In 2007, Odemiş et al^[2]^ reported that the incidence of PBNHL in patients with malignant cholangiocarcinoma was 0.6%, and PBNHL accounted for 0.4% of extranodal non-Hodgkin's lymphoma and only 0.016% of all non-Hodgkin's lymphoma cases. Hepatitis B virus, hepatitis C virus, human immunodeficiency virus Epstein-Barr virus infection, elevated lactate dehydrogenase (LDH), or low immunity are associated with the development of PBNHL.^[3–5]^ This paper reports a case of PBNHL that appeared like hilar cholangiocarcinoma.

## 2 Case presentation

A 59-year-old woman was admitted to our hospital with a 3-day history of jaundice associated with generalized itching, pale stools, and dark urine. There was no history of nausea or vomiting. Oil aversion, anorexia, abdominal pain, or fever was also absent. History of weight loss was present which was about 5 kg in the past 3 months. Past medical history was unremarkable. Laboratory tests showed elevated total bilirubin, direct bilirubin, alkaline phosphatase, and LDH. Routine blood tests were regular. Immunoglobulin M antibody to Epstein-Barr virus (EBV-IgM) was positive. Immune-related laboratory tests revealed immunoglobulin G4 level was average. Tumor marker showed elevated carbohydrate antigen 19–9 (CA 19–9) and normal alpha-fetoprotein and carcinoembryonic antigen.

**Figure 1:**
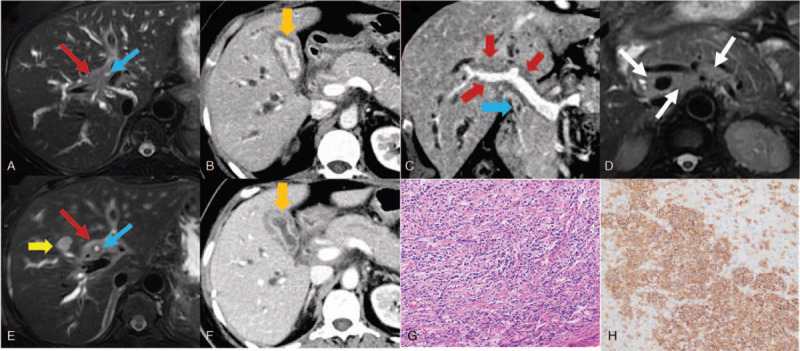
A 55-year-old woman with pathology confirmed primary hepatobiliary lymphoma. A–D, The initial abdominal images of the patient. A, Axial T2-weighted MRI showed intermediate homogeneous intensity mass surrounding the hilar bile duct (red arrow) with dilated intrahepatic ducts. B, Axial contrast-enhanced CT demonstrated thickening gallbladder wall with marked enhancement (yellow arrow). C, Coronal reconstruction contrast-enhanced CT showed blurred fat space around the portal vein and hepatic artery in hepatic hilum without lumen stenosis (red arrow). And the diffuse thickening wall of CBD was noted (blue arrow). D, Axial T2-weighted MRI showed lymphadenopathy (white arrows) around the celiac trunk and portal vein. Figures E and F were the following-up images 1 month later without any antitumor therapy. E, Axial T2-weighted MRI showed that the common hepatic duct diminished in stenosis than the initial image (blue arrow), but a new parenchyma lesion was observed (yellow arrow). F, Axial contrast-enhanced CT demonstrated a thinner gallbladder wall (yellow arrow) and enlarger cavity. G, HE staining showed medium-large lymphoid cell infiltration in the gallbladder wall. H, The tumor cell membranes were positive for CD20. CBD = common bile duct, CT = computed tomography, HE = hematoxylin-eosin, MRI = magnetic resonance imaging.

Initial computed tomography (CT) and magnetic resonance imaging images showed diffuse thickening wall of bile ducts (including common bile duct, common hepatic duct, and intrahepatic ducts) with corresponding lumen stenosis and blurred fat space around portal vein. Thickening of the gallbladder wall was also noted. All of these lesions above exhibited significantly diffusion restricted and moderately homogeneous enhancement. Lymphadenopathy was found around the celiac trunk and its branches. The spleen was normal (Fig. 1 A–D). One month after the patient's first examination, without any antitumor treatment, both computed tomography and magnetic resonance imaging images showed diminished stenosis of common bile duct and left hepatic duct, but a new mass in segment IV of liver was observed (Fig. 1 E, F).

The diagnosis of hilar cholangiocarcinoma was first considered because it was combined with elevated tumor marker CA19–9 and images showing gallstones with cholecystitis.

**Figure 2:**
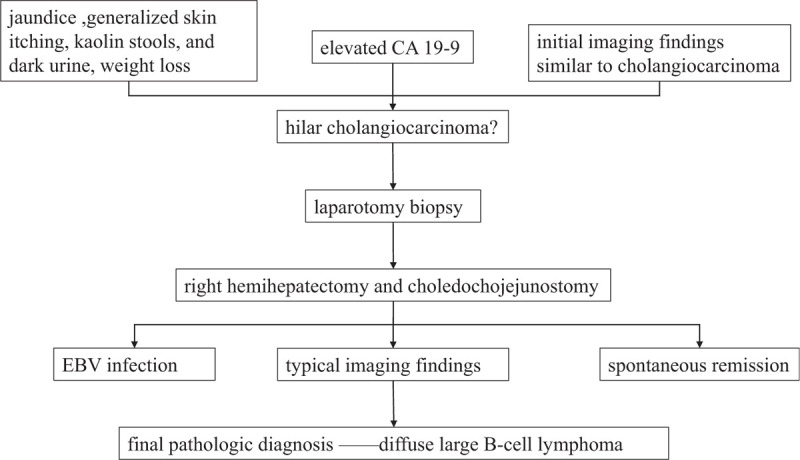
Important milestones related to the diagnoses and interventions.

One month later, the patient and her family requested to clarify the histologic diagnosis by laparotomy biopsy. Because the biopsy result could not be defined during operation, then right hemihepatectomy and choledochojejunostomy were performed. During the operation, significant contracture of the first porta hepatis, thickening of the hepatoduodenal ligament and the wall of the right hepatic duct, stenosis of the right posterior bile duct was observed. Moreover, lymphadenopathy at the portal vein, antrum, and proper hepatic artery could be found. The postoperative pathology report showed atypical cells in group 8 of the lymph nodes were observed. Furthermore, the surgical margin was negative. The immunohistochemistry of bile duct tissue suggested that FOXPI (80%), Ki-67 (70%), Bcl-6 (60%), MUM-1 (70%), c-myc (3%), CD38 Kappa, CD43, CD20, and CD79a were positive, and Lambda and Bcl-2 were negative (Fig. 1 G). So, the final pathologic diagnosis was diffuse large B-cell lymphoma, stage IV. Unfortunately, the patient died 2 months later after the operation due to disease progression. Important milestones related to the diagnoses and interventions are listed in Figure 2.

## 3 Discussion

PBNHL is a rare type of diffuse large B-cell lymphoma. Clinical manifestations are jaundice, epigastric pain, fever, weight loss, and abdominal mass. Biliary obstruction is a sign of poor prognosis.^[2,6]^ Imaging features of PBNHL reported so far are very limited, and mainly include diffuse space-occupying lesions or irregular thickening of the bile duct, mild biliary stricture, and secondary biliary dilatation.^[2,7,8]^ The diagnosis of lymphoma is mainly based on histopathology, immunohistochemistry, combined with clinical data and imaging findings. Positron emission tomography-computed tomography can be used to determine the extent of tumor invasion.^[9]^ However, clinicians may misdiagnose PBNHL as cholangiocarcinoma or immunoglobulin G4-associated cholangitis (IAC).

IAC patients have elevated serum and bile immunoglobulin G4 levels with the imaging feature of homogeneous progressive enhancement of the symmetric thickening wall (including the stenotic area and nonstenotic area). The most common location of IAC is inferior portion of the common bile duct. IAC is frequently accompanied by pancreatic inflammation.^[10,11]^ Cholangiocarcinoma presents that the tumor marker CA19–9 increases significantly, and the extent of lesion is more localized, and bile duct stenosis is severe and stiff. The biliary stricture in lymphoma is softer than cholangiocarcinoma. The patient without any antitumor treatment, the imaging showed relief of some biliary strictures, which has been reported in lymphoma, but it has not been reported in cholangiocarcinoma.^[12–14]^

However, establishing the diagnosis of PBNHL can be challenging by relying only on imaging and clinical manifestations. Even for biopsy, the results are often misdiagnosed as chronic nonspecific inflammation and other malignant tumors. So, surgery is inevitable to obtain enough tissue and achieve an accurate diagnosis.^[15,16]^ At the same time, due to the rarity of PBNHL, unified treatment plan is uncertain. But it is believed that once the diagnosis is confirmed, chemotherapy is the basic and necessary treatment. Immunochemotherapy with rituximab plus cyclophosphamide, doxorubicin, vincristine, and prednisone or similar regimens is generally used.^[7,17]^ And if biliary obstruction occurs, the biliary diversion is required.^[2]^ Radiotherapy has been used in some patients and can be used for patients with residual disease after primary chemotherapy, or to relieve pain. However, whether radiation therapy is an essential treatment remains to be further studied because of the rarity of the disease.^[16,18]^ Besides PBNHL also lacks studies to definite prognosis. The overall survival can be less than 1 year due to disease progression caused by adverse effects of surgery or the lack of chemotherapy.^[1,19]^

## 4 Conclusion

Although it is difficult to diagnose PBNHL preoperatively, diagnosis of PBNHL should be considered when the bile duct is diffusely thickened without stenosis and the surrounding fat space is blurred. At the same time, factors such as infection with Epstein-Barr virus, elevated LDH, typical imaging findings, and spontaneous remission of the patient's condition are more suggestive of this disease.

## Acknowledgments

The authors wish to thank Jannatul Maoya Bashanti for the language polishing and Paperpal Preflight Service for writing assistance.

## Author contributions

**Conceptualization:** Yun Mao.

**Data curation:** Jiamei Wu, Jing Zhang.

**Project administration:** Yun Mao, Yin Zhou.

**Resources:** Yin Zhou, Qingshu Li.

**Supervision:** Yun Mao, Yin Zhou, Qingshu Li.

**Writing – original draft:** Jiamei Wu.

**Writing – review & editing:** Jiamei Wu, Yun Mao.

## References

[1] Nguyen GK. Primary extranodal non-Hodgkin's lymphoma of the extrahepatic bile ducts. Report of a case. Cancer 1982;50:2218–22. 7127262 10.1002/1097-0142(19821115)50:10<2218::aid-cncr2820501041>3.0.co;2-4

[2] Odemiş B Parlak E Başar O. Biliary tract obstruction secondary to malignant lymphoma: experience at a referral center. Dig Dis Sci 2007;52:2323–32. 17406815 10.1007/s10620-007-9786-4

[3] Noronha V Shafi NQ Obando JA. Primary non-Hodgkin's lymphoma of the liver. Crit Rev Oncol/Hematol 2005;53:199–207. 10.1016/j.critrevonc.2004.10.010 15718146

[4] Emile JF Azoulay D Gornet JM. Primary non-Hodgkin's lymphomas of the liver with nodular and diffuse infiltration patterns have different prognoses. Ann Oncol 2001;12:1005–10. 11521784 10.1023/a:1011131930409

[5] Dlouhy I Filella X Rovira J. High serum levels of soluble interleukin-2 receptor (sIL2-R), interleukin-6 (IL-6) and tumor necrosis factor alpha (TNF) are associated with adverse clinical features and predict poor outcome in diffuse large B-cell lymphoma. Leuk Res 2017;59:20–5. 28544905 10.1016/j.leukres.2017.05.014

[6] Khozeimeh N Bhatti T Ponsky TA. Primary Non-Hodgkin's lymphoma of the extrahepatic bile duct. J Gastrointestinal Cancer 2012;43 suppl 1:S46–9. 10.1007/s12029-011-9353-2 22198638

[7] Joo YE Park CH Lee WS. Primary non-Hodgkin's lymphoma of the common bile duct presenting as obstructive jaundice. J Gastroenterology 2004;39:692–6. 10.1007/s00535-004-1367-0 15293142

[8] Zakaria A Al-Obeidi S Daradkeh S. Primary non-Hodgkin's lymphoma of the common bile duct: a case report and literature review. Asian J Surg 2017;40:81–7. 24239512 10.1016/j.asjsur.2013.09.009

[9] Liu Y Barta SK. Diffuse large B-cell lymphoma: 2019 update on diagnosis, risk stratification, and treatment. Am J Hematol 2019;94:604–16. 30859597 10.1002/ajh.25460

[10] Itoh S Nagasaka T Suzuki K. Lymphoplasmacytic sclerosing cholangitis: assessment of clinical, CT, and pathological findings. Clin Radiol 2009;64:1104–14. 19822244 10.1016/j.crad.2009.07.006

[11] Li J Zhao C Shen Y. Autoimmune cholangitis and cholangiocarcinoma. J Gastroenterol Hepatol 2012;27:1783–9. 23034143 10.1111/j.1440-1746.2012.07287.x

[12] Patel AH Harnois DM Klee GG. The utility of CA 19-9 in the diagnoses of cholangiocarcinoma in patients without primary sclerosing cholangitis. Am J Gastroenterol 2000;95:204–7. 10638584 10.1111/j.1572-0241.2000.01685.x

[13] Manfredi R Barbaro B Masselli G. Magnetic resonance imaging of cholangiocarcinoma. Semin Liver Dis 2004;24:155–64. 15192788 10.1055/s-2004-828892

[14] Choi BI Lee JM Han JK. Imaging of intrahepatic and hilar cholangiocarcinoma. Abdom Imaging 2004;29:548–57. 15185025 10.1007/s00261-004-0188-1

[15] Kosuge T Makuuchi M Ozaki H. Primary lymphoma of the common bile duct. Hepatogastroenterology 1991;38:235–8. 1937362

[16] Maymind M Mergelas JE Seibert DG. Primary non-Hodgkin's lymphoma of the common bile duct. Am J Gastroenterol 1997;92:1543–6. 9317083

[17] Luigiano C Ferrara F Fabbri C. Primary lymphoma of the common bile duct presenting with acute pancreatitis and cholangitis. Endoscopy 2010;42 suppl 2:E265–6. 20931475 10.1055/s-0030-1255766

[18] Ravindra KV Stringer MD Prasad KR. Non-Hodgkin lymphoma presenting with obstructive jaundice. Br J Surg 2003;90:845–9. 12854111 10.1002/bjs.4119

[19] Oda I Inui N Onodera Y. [An autopsy case of primary non-Hodgkin lymphoma of the extrahepatic bile duct]. Nihon Shokakibyo Gakkai Zasshi 1999;96:418–22. 10332205

